# Impact of Physical and Chemical Modification of the Surface of Porous Al_2_O_3_ Ceramic Membranes on the Quality of Transferred HSMG^®^ and CVD Graphene

**DOI:** 10.3390/membranes13030319

**Published:** 2023-03-09

**Authors:** Aleksandra Bednarek, Konrad Dybowski, Grzegorz Romaniak, Jacek Grabarczyk, Witold Kaczorowski, Anna Sobczyk-Guzenda

**Affiliations:** Institute of Materials Science and Engineering, Faculty of Mechanical Engineering, Lodz University of Technology, 1/15 Stefanowskiego St., 90-924 Lodz, Poland

**Keywords:** HSMG^®^ graphene, CVD graphene, graphene transfer, porous ceramics, Al_2_O_3_, surface modification

## Abstract

Graphene transfer onto ceramics, like Si/SiO_2_, is well-developed and described in the literature. However, it is problematic for other ceramic materials (e.g., Al_2_O_3_ and ZrO_2_), especially porous ones. In this case, it is mainly due to poor adhesion to the substrate, resulting in strong degradation of the graphene. For these reasons, the research topic of this study was undertaken. This article presents research on the development of the methodology of graphene transfer onto ceramic Al_2_O_3_ surfaces. Polycrystalline graphene chemical vapour deposition (CVD) monolayer and quasimonocrystalline high-strength metallurgical graphene (HSMG^®^) synthesised on liquid copper were used. When developing the transfer methodology, the focus was on solving the problem of graphene adhesion to the surface of this type of ceramic, and thus reducing the degree of graphene deterioration at the stage of producing a ceramic–graphene composite, which stands in the way of its practical use. Plasma and chemical ceramic surface modification were applied to change its hydrophobicity, and thus to improve the adhesion between the graphene and ceramic. The modification included the use of dielectric barrier discharge (DBD) plasma, oxygen plasma (RF PACVD method - Radio Frequency Plasma Assisted Chemical Vapour Deposition), and hydrofluoric acid treatment. Changes in surface properties caused by the modifications were determined by measuring the contact angle and (in the case of chemical modification) measuring the degree of surface development. The effectiveness of the applied surface preparation methodology was evaluated based on the damage degree of CVD and HSMG^®^ graphene layer transferred onto modified Al_2_O_3_ using optical microscopy and Raman spectroscopy. The best average I_D_/I_G_ ratio for the transferred HSMG^®^ graphene was obtained after oxygen plasma modification (0.63 ± 0.18) and for CVD, graphene DBD plasma was the most appropriate method (0.17 ± 0.09). The total area of graphene defects after transfer to Al_2_O_3_ was the smallest for HSMG^®^ graphene after modification with O_2_ plasma (0.251 mm^2^/cm^2^), and for CVD graphene after surface modification with DBD plasma (0.083 mm^2^/cm^2^).

## 1. Introduction

Graphene, thanks to its unique mechanical, thermal, and electrical properties, is widely known as an innovative material of the future [1,2,3,4,5]. Recent intensive research in the field of graphene materials revealed challenges in maintaining a high quality of graphene after transfer from growth substrates to another material. This one-atom-thick layer loses its properties when being torn or dirty, and thus, the possibility of practical applications is limited.

The methods of production of large-area graphene include synthesis on solid metal by chemical vapour deposition (CVD) [6], synthesis on liquid metals by CVD [7,8,9,10], and synthesis by metallurgical methods on liquid copper (HSMG^®^) [11,12,13]. All these methods demand the use of an effective procedure to transfer graphene on a target substrate to be able to use graphene in further research and applications.

Many literature reports concern the use of graphene in filtration, but these are solutions based mainly on polymer membranes [14,15,16,17,18,19,20,21,22,23]. There are few reports on the use of ceramic membranes for this purpose. Comparing these two supports for graphene, it can be concluded that membranes based on a ceramic substrate will show higher mechanical properties, excellent thermal stability, and chemical resistance. So from a practical point of view, there are many applications where graphene–polymer membranes cannot be used. Ceramic membranes are usually prepared from metal oxides such as alumina, silica, zirconium, titanium, etc., or natural materials (e.g., natural clay and phosphate) [24,25,26,27,28]. The problem with using these carrier materials to build composite membranes with graphene is the adhesion between graphene and ceramics. Although this is a fundamental problem in using this type of material, little attention has been paid to this issue so far.

Most of the graphene transfer methods presented in the literature refer to procedures where quartz (SiO_2_) is used as the target ceramic substrate. Silicon itself or its oxide are widely used substrate materials for studying the effectiveness of improved transfer methods. The lack of use of other ceramic materials for this purpose is explained by their surface morphology and physicochemical properties, by which graphene shows poor adhesion to ceramics and is damaged during the removal of the supporting polymer [29,30].

The most commonly used methods of graphene transfer consist of a few steps, with the most important steps being: graphene straightening, polymer temporary layer application on graphene, growth substrate removal, cleaning, transfer on the target substrate, and polymer removal. Removal of the growth substrate can be conducted by etching of metal or by separating graphene via electrochemical delamination, where due to the voltage applied, graphene is negatively polarised and hydrogen is emitted between graphene and the metallic substrate, and hence graphene peeling is possible. This method is more accurate for thicker growth substrates and the obtained graphene is cleaner [30,31,32,33,34].

Other transfer methods like polymer-free transfer, in which the target substrate is pressed to graphene on a growth substrate are used for thin and elastic materials like porous polycarbonate. It is not adequate for rigid ceramic materials [32].

Ceramic membranes are more thermally, chemically, and mechanically stable than polymeric ones, which makes them more durable. The nature of these materials makes them hydrophilic due to the presence of hydroxyl (-OH) groups on the surface. Commercial membranes are modified by hydrophobization for use in separation processes. This effect would also be possible by covering them with a layer of graphene [35,36,37]. Oxygen plasma [38], argon etching [39], low-temperature H_2_, N_2_, and O_2_ plasma [40] were proven to be effective in improving the hydrophilic properties of the ceramic surface, and thus facilitating the transfer of graphene. Attempts were also made to soften the PMMA layer for better adhesion between the graphene and ceramic substrate to improve graphene transfer quality [29].

Bearing in mind, on the one hand, the potential benefits of using graphene–ceramic composite membranes, and on the other hand, the problems with transferring the graphene layer to ceramics, the purpose of this work were focused on this basis. The work aimed to determine the effect of chemical and physical modifications of the surface of the ceramic porous membrane on the quality of the transferred graphene. Large-area graphene produced by the CVD and HSMG^®^ methods was used for the study. The surface of Al_2_O_3_ ceramics was modified by means of barrier plasma, oxygen plasma, and etching with hydrofluoric acid. The surface changes were assessed based on FTIR (Fourier Transform Infrared Spectroscopy) tests and the wetting angle measurement. The quality of the transferred graphene layers was evaluated based on optical microscopy and Raman spectroscopy. On this basis, the relationship between the state of the ceramic surface and the obtained quality of graphene transferred to its surface was demonstrated. We also determined which of the surface modification methods is optimal for obtaining the best quality transferred graphene layer.

## 2. Materials and Methods

### 2.1. Materials

Porous oxide ceramic Al_2_O_3_ discs with a diameter of 47 mm and pore size <0.1 µm (Cobra Technologies B.V., Rijssen, Netherlands) were used for surface modification and graphene transfer. CVD graphene synthesized on solid copper and HSMG^®^ graphene synthesized on liquid copper were used for transfer onto ceramics.

### 2.2. HSMG^®^ Synthesis

HSMG^®^ synthesis was conducted according to the patent [41]. The process consists of the following steps:

1. Preparation of nickel foils with a thickness of 0.1 mm and dimensions of 220 × 120 mm^2^.
2. Galvanic coating of the Ni foil with a layer of copper with a thickness of 100 µm in a solution of copper sulphate pentahydrate (CuSO_4_·5H_2_O), at a current density of 0.02 A/cm^2^ for 6 h.
3. Graphene synthesis on a Cu-Ni bimetallic substrate in a vacuum generator SuperCarb, Seco/Warwick SA, Świebodzin, Poland, according to the procedure:
  a. Heating in vacuum (10 Pa pressure) up to 1060 °C.
  b. Carburizing in an atmosphere of acetylene, ethylene, and hydrogen (2:2:1), at a flow rate of 4.0 L/min acetylene and ethylene, and 2.0 L/min hydrogen, consisting of four alternating stages of carbonation and annealing, 5 s of gas dosing/15 min diffusion.
  c. Heating in an atmosphere of argon and hydrogen to a temperature above the copper melting point (1100 °C) under a pressure of 2 kPa followed by a 5 min soak.
  d. Cooling the batch to ambient temperature under the same conditions.

### 2.3. CVD Graphene Synthesis on Solid Copper Substrate

The CVD graphene synthesis process on copper solid-state foils was conducted by creating optimal conditions for carburizing in the SuperCarb vacuum generator. An acetylene, ethylene, and hydrogen (2:2:1) gas mixture was used with gas flow rates of 4.0 L/min for acetylene and ethylene and 2.0 L/min for hydrogen. Graphene was synthesized on copper foils 0.1 mm thick and 50 × 50 mm^2^ in size. The foils were previously washed in an ultrasonic cleaner in isopropanol and then in acetone.

The process of producing CVD graphene was as follows:

1. Heating in vacuum (10 Pa) to a temperature of 1000 °C,
2. Carburizing in an acetylene–ethylene–hydrogen atmosphere for 1 min.
3. Heating in a vacuum at 1000 °C for 5 min.
4. Carburizing in an acetylene–ethylene–hydrogen atmosphere for 0.5 min.
5. Cooling in vacuum to ambient temperature.

### 2.4. Separation of HSMG^®^ and CVD Graphene from the Growth Substrate

Graphene was separated from the growth substrate using the electrochemical delamination method, which was preceded by drop/blade coating of graphene by a one or two-layer carrier polymer. A 0.05 M solution of polymethyl methacrylate (46 mg of PMMA (996,000 g/mol, Aldrich Chemistry Company Burlington, MA, USA) dissolved in 1 mL of chlorobenzene Chempur Piekary Śląskie, Poland) was used in the amount of 40 μL of solution per 1 cm^2^ of graphene. The polymer was dried at a temperature of c.a. 40 °C for 30 min. The double support layer consisted of an additional top layer of poly(dimethylsiloxane), produced by applying about 20 mg of liquid PDMS per cm^2^ of graphene. The polymer was prepared from a silicone elastomer base and hardener SYLGARD^®^ 184 in a ratio of 10:1. The drying time of the PDMS layer at 60 °C was 60 min. After that, the growth substrate with graphene and polymer was placed in the grip in a 0.5 M NaOH solution so that only its edge was immersed in the electrolyte. The process of graphene delamination was started by applying a constant voltage in the range of 2.5–5 V, causing hydrogen generation between the graphene and growth substrate (cathode). The progress of graphene separation was carried out at a constant speed of 0.01–0.05 mm/s set in the program so that the graphene separation line was at the level of the liquid level. The graphene was then washed twice in distilled water and air dried. Graphene quality was tested by measuring its resistance using the UNI-T UT70B multimeter, UNI-TREND Technology, China, with needle probes.

### 2.5. Graphene Transfer to a Target Substrate

The reference point in the development of the graphene transfer methodology to alumina ceramics (Al_2_O_3_) was quartz (SiO_2_). Transfer on quartz is less problematic thus methods presented in the literature were adapted for the needs of graphene transfer to a porous aluminium oxide substrate. However, porous Al_2_O_3_ is such a different material that the adoption of the 1:1 transfer methodology resulted in poor quality graphene, the main reason being insufficient adhesion of graphene to porous ceramics. The standard wet transfer procedure with the elimination of the polymer in boiling acetone vapour, which works well in the case of quartz, did not work in this case. Attempts were made to increase the adhesion of graphene to ceramics by applying 2 mL of a 75% acetone/water solution on the sample before placing in it the bath [29]. However, loading the foil with acetone drops did not solve the problem of poor adhesion, so it was decided to carry out chemical and plasma modification of the substrate, the purpose of which was to improve the adhesion of graphene to ceramics.

### 2.6. Increasing the Hydrophilicity of the Ceramic Surface

#### 2.6.1. Surface Etching with Dielectric Barrier Discharge (DBD) Plasma

The ceramics were treated with low-temperature plasma under atmospheric pressure, generated as a result of a dielectric-barrier discharge (DBD). The system consists of two metal discharge electrodes covered with a dielectric, the so-called dielectric barriers, separated from each other by a 2-millimetre discharge gap. After the system is powered by a high-voltage generator with a frequency of approx. 80 kHz, plasma is generated in the space between the electrodes.

In order to improve the adhesion of graphene to ceramics, an attempt was made to change the properties of its surface by creating a barrier discharge powered with a voltage of 15 kV and a frequency of 80 kHz. The time of exposure of the samples to the plasma thus formed was 1 min.

#### 2.6.2. Modification of the Surface with Oxygen Plasma by the RF PACVD Method

In order to increase the adhesion between the transferred graphene and the target ceramic substrate, an attempt was also made to modify the Al_2_O_3_ surface with oxygen plasma. For this purpose, the RF PACVD (Radio Frequency Plasma Assisted Chemical Vapour Deposition) method was used. The main components of the RF PACVD plasma surface modification equipment are: a working chamber with an HF (high-frequency) electrode, a diffusion pump, a gas dosing system, a pressure measurement system, a radio frequency (13.56 MHz) generator, and a pyrometer for temperature control. The process parameters are shown in Table 1.

#### 2.6.3. Etching Ceramics with Hydrofluoric Acid

Chemical etching of the ceramics with an aqueous solution of hydrofluoric acid (HF; Aldrich Chemistry Company Burlington, MA, USA) was also used to increase the adhesion of graphene to the surface. Solutions of two concentrations were used: 5 and 9.5% HF; pickling time 15 min.

### 2.7. Changes in Hydrophilicity of the Modified Ceramics

The assessment of the change in wettability of the ceramic surface with distilled water after the modifications was made using a Krüss DSA10 goniometer, Hamburg, Germany. The contact angles were determined based on photos of liquid drops with a volume of 0.8 μL placed on the surface of the samples, taken with a camera set in the axis perpendicular to the surface of the sample. Investigations on non-porous Al_2_O_3_ samples were performed.

### 2.8. Evaluation of Surface Roughness of Modified Ceramic

To measure the change in ceramic surface roughness after etching with hydrofluoric acid (HF), a contact profilometer HOMMEL TESTER T1000was used, supported by the Turbo Datawin NT 1.34 software, both from JENOPTIK Industrial metrology, Villingen-Schwenningen, Germany, enabling the determination of surface roughness and waviness parameters based on the obtained etching profiles. Based on the Ra measurement results, the effect of HF acid concentration on the change in the surface topography of the tested samples was determined.

### 2.9. Study of the Chemical Structure (FTIR Spectroscopy) of the Ceramic Surface before and after Modification

The chemical structure was determined by using Fourier-transform infrared spectroscopy (FTIR) using a Thermo Scientific iS50 Spectrometer, Waltham, MA, USA, with a spectral range from 4000 to 400 cm^−1^. Spectra were registered with a resolution of 4 cm^−1^ with the use of a DTGS detector. The measurements were made in a reflection mode, with the use of Sequelle DRIFT mode, working at a reflection angle equal to 20 degrees. In the case of each material, data from 128 scans were collected in order to construct a single spectrum.

### 2.10. Macro and Microscopic Graphene Transfer Quality Evaluation

In order to detect large defects on the surface, the transferred graphene layers were observed at low magnification using a Nikon AZ, Tokyo, Japan stereomicroscope with NIS-Elements software for automatic image registration and processing.

A Nikon Eclipse MA200, Tokyo, Japan light microscope, equipped with NIS-Elements image analysis software, was used to accurately assess the defects in the surface of the transferred graphene in micro-areas. This type of microscope was used because, due to the strong representation of the topography of the ceramic surface, the analysis of the continuity of the graphene layer using a scanning electron microscope, both in the secondary electron (SE) and AEE mode (imaging of differences in surface electrical conductivity), turned out to be ambiguous. ImageJ software, LOCI, Madison, WI, USA, was used to perform statistical evaluation of graphene defects after transfer onto a porous ceramic substrate. An area of 1 cm^2^ was analysed each time. In this way, the share of defects in the total area of the analysed sample was calculated.

### 2.11. Qualitative Assessment of Graphene after Transfer to Al_2_O_3_ Ceramics Using Raman Spectroscopy

The Raman spectra and maps were carried out with an InVia Raman microscope (Renishaw plc, Gloucestershire, UK) system. The graphene surfaces were exanimated using a 532 nm laser with a 50× objective lens (Carl-Zeiss, Jena, Germany). The laser power at the sample was restricted to 4 mW. For Raman maps, map image acquisition mapping was applied in the wavenumber range of 900 to 3200 cm^−1^. Two-dimensional Raman maps were collected from 50 × 50 μm areas with 5 μm spatial resolution. All the collected Raman spectra and maps were preprocessed using WiRE 5.5 software (Renishaw plc, Gloucestershire, UK).

## 3. Results and Discussion

### 3.1. Ceramics Surface Hydrophilic Properties Modification

#### 3.1.1. Evaluation of Ceramics Surface Roughness

**Figure 1:**
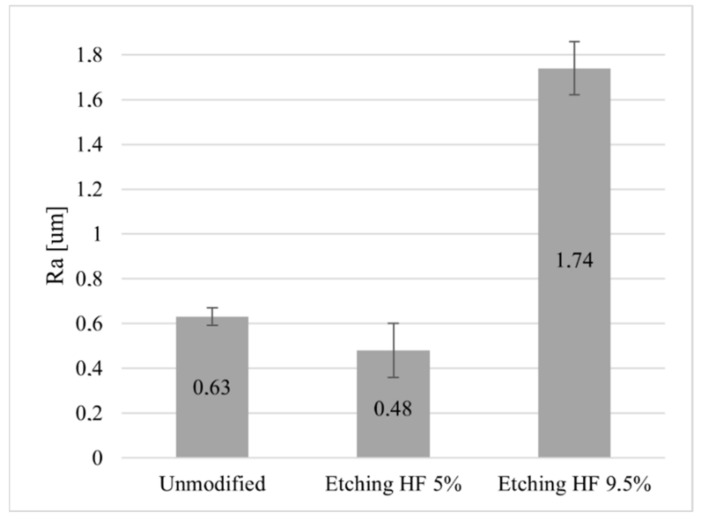
Change in Ra value of chemically modified Al_2_O_3_ ceramic surface.

To increase the contact area between the surface layer of ceramics and graphene as a result of etching the ceramic surface with 5% and 9.5% hydrofluoric acid (HF) solutions, an analysis of the Ra value of the surface of Al_2_O_3_ ceramics before and after chemical modification was carried out. The analysis of the data presented in Figure 1 shows that etching the surface of the ceramics Al_2_O_3_ with a 5% HF acid solution resulted in the levelling of its surface unevenness, manifested by a decrease in the value of the Ra parameter by 0.15 µm compared to its initial state. On the other hand, the almost 2-fold increase in the acid concentration caused a drastic increase in the surface roughness of the Al_2_O_3_ ceramics by more than 1µm compared to its state before the modification.

#### 3.1.2. Ceramic Surface Wettability Analysis

**Figure 2:**
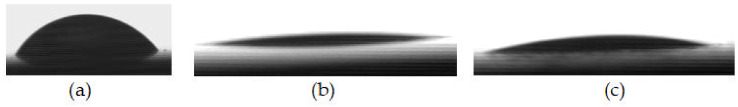
Change in a water droplet shape on the Al_2_O_3_ ceramic surface: (**a**) unmodified, (**b**) plasma modification, (**c**) chemical modification.

Investigations of the ceramics’ wettability with the use of distilled water showed that corundum oxide (Al_2_O_3_) has weak hydrophilic properties, consistent with the literature data [42]. After treatment with plasma with barrier discharges and oxygen plasma, the surface became superhydrophilic; due to this, the measurement of the contact angle using the drop geometry method is burdened with a large error (Figure 2b). The improvement of the wettability of the ceramic surface was also obtained as a result of chemical modification. However, the obtained effect of improving the hydrophilicity of the surface after acid treatment was weaker than as a result of plasma. Furthermore, the results show that increasing the acid concentration 2-fold did not significantly increase this effect. The results of the contact angle measurements with distilled water of pure and modified ceramic samples are presented in Table 2, and Figure 2 shows the change in the appearance of water droplets on the ceramic surface after the applied modifications.

Comparing the roughness results obtained with the results of the surface wettability test, it can be seen that the improvement of the hydrophilicity of the Al_2_O_3_ ceramics as a result of the chemical modification is ambiguous, because with a lower concentration of HF acid, its surface was smoothed, and with a higher concentration, its roughness was significantly increased. According to the literature [43], regardless of the concentration of HF acid used, the etching of ceramics should improve its wettability as a result of surface development. Due to the lack of differences in the wetting results, a chemical modification with a lower concentration of the acid solution (5%) was used for further research because it gave a smaller parameter Ra. The high roughness obtained after etching with the 9.5% solution could have caused damage to the graphene layers after the transfer.

### 3.2. Chemical Analysis of the Effect of Surface Modification of Porous Al_2_O_3_ Ceramics by DBD, Oxygen Plasma, and HF

**Figure 3:**
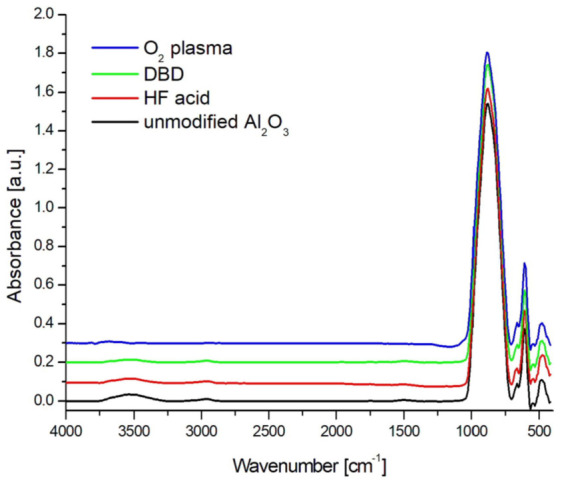
FTIR spectra for unmodified and modified ceramics using 5% HF acid, DBD, and O_2_ plasma in the range of 4000–400 cm^−1^.

Figure 3 presents the FTIR spectrum of oxygen plasma RF PECVD-, DBD-, and 5% HF acid-modified ceramics and unmodified ceramics.

In the spectrum of unmodified and modified ceramics presented in Figure 3, there were characteristic bands typical for α-Al_2_O_3_ originating from Al-O stretching vibrations at wave numbers 467, 600, and 667 cm^−1^. There was also a wide band in the range of wavenumbers from 700 to 1030 cm^−1^, which is associated with deformation vibrations of Al-OH bonds. In the wavenumber range of 3300–3700 cm^−1^, there was a wide band originating from O-H bond stretching vibrations [44]. However, the range below 3600 cm^−1^ belongs to the vibrations physically connected with the water surface, in which hydrogen bonds are formed between the hydroxyl groups [45]. On the other hand, the peaks at higher wavenumber values come from isolated hydroxyl groups chemically bound to the Al_2_O_3_ surface, which are strongly bound to the ceramic surface. In the unmodified ceramics, there were traces of organic impurities, which were revealed in the FTIR spectrum in the form of asymmetric and symmetrical C-H stretching vibrations in the range of 3000–2800 cm^−1^ [46].

The waveform of spectra for HF acid- and DBD-modified ceramics was identical to that for unmodified ceramics. The only significant difference was in the range of 3300–3700 cm^−1^ and was associated with a smaller amount of hydroxyl groups chemically and physically bonded to the surface. The shape of the spectra did not change.

**Figure 4:**
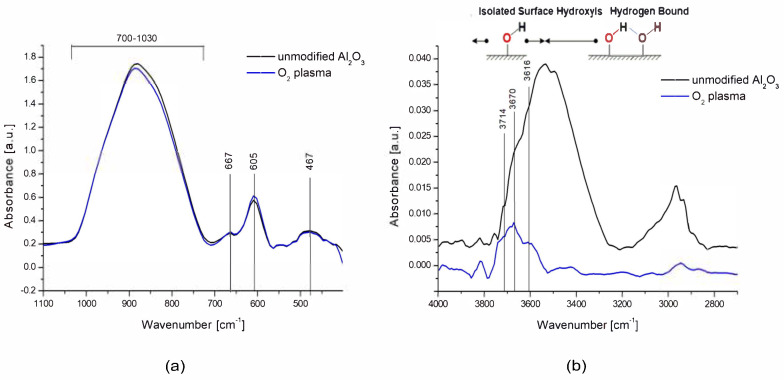
FTIR spectra for unmodified and modified O_2_ plasma ceramics in the ranges 1100–400 cm^−1^ (**a**) and 4000–2700 cm^−1^ (**b**).

Modification of the ceramic surface in low-temperature oxygen plasma resulted in the removal of most of the hydroxyl groups derived from molecular water physically bonded to the tested surface which is presented in Figure 4a. In the range of 3600–3800 cm^−1^, three peaks at wave numbers 3714, 3670, and 3615 cm^−1^ remained well separated, originating from chemically bonded, isolated hydroxyl groups, which, according to the theory of Busce and Tsyganenko, can originate from bridged and triple bridged oxygen, as well as terminal Al-OH groups. Some of these maxima could also be isolated in the spectra of ceramics before modification because they were components of a wide band coming from the adsorbed molecular water [45].

**Figure 5:**
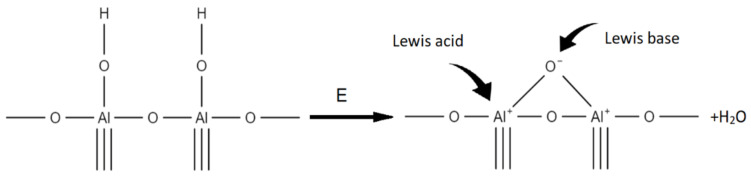
Oxygen bridge creation during plasma treatment according to Lewis principle.

The chemically bound water on the aluminium oxide surface is considered as an interaction between the electron-accepting aluminium ion (Lewis acid) and the hydroxyl donor ion, which is the so-called Lewis principle. On the other hand, hydroxyl groups behave as Bronsted acid sites. However, high-energy plasma surface cleaning can cause two adjacent OH ions on the alumina surface to form an oxygen bridge, which, however, is the Lewis acid active site, as shown in the diagram below (Figure 5). Such a system exhibits chemical activity and is able to bind both water and other chemical structures [46,47]. This process takes place with the release of water, which is removed from the reaction environment during the modification taking place in a vacuum.

Moreover, surface modification in plasma also cleans/etches the surface of organic impurities. The band in the range of 3000–2800 cm^−1^ disappeared almost completely.

In the range of 1030–400 cm^−1^, there was also a slight increase in the intensity of the peaks at the wave numbers 600 and 657 cm^−1^, which originate from the Al-O bonds characteristic of the alumina structure, which means that the surfaces may have undergone additional oxidation (to a small extent) which is presented in Figure 4b.

### 3.3. Macro and Microscopic Assessment of Graphene HSMG^®^ and CVD Quality after Transfer onto Surface-Modified Ceramics

**Figure 6:**
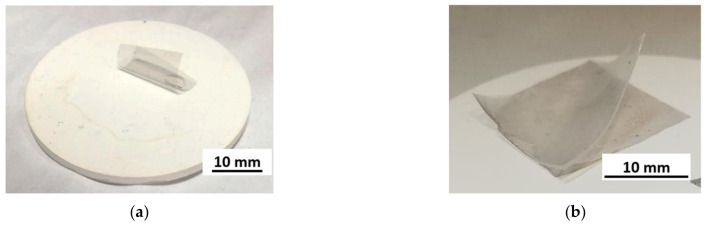
Al_2_O_3_ ceramic surface during HSMG^®^ graphene transfer sample: (**a**) without surface modification—lack of graphene–PMMA adhesion to the surface; (**b**) with surface modification—graphene adheres to the surface of ceramics; delamination of an additional PDMS layer during removal of the PMMA support layer in alcohol vapour.

Attempts to transfer graphene to aluminium oxide ceramics without modifying its surface have not yielded a positive result. Graphene, as seen in Figure 6a, is not able to stay on ceramics. PMMA–graphene foil rolls up and graphene does not adhere to the surface. Only the application of surface modification makes it possible to transfer graphene to Al_2_O_3_ ceramics. The picture (Figure 6b) of the graphene surface after transfer to the modified ceramic show that the use of the technique to improve hydrophilicity in combination with an additional PDMS support layer allowed the transfer of a large surface area of the continuous graphene layer.

**Figure 7:**
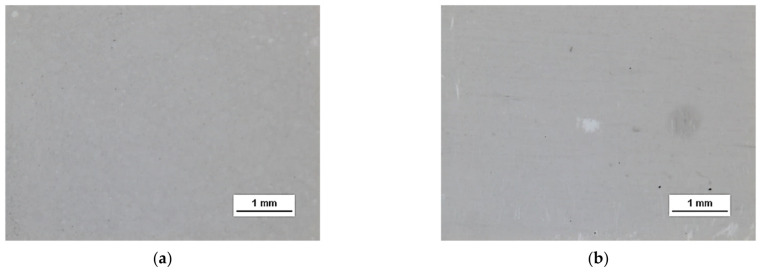
Microscope images of the surface of graphene: (**a**) HSMG^®^ and (**b**) CVD, transferred to Al_2_O_3_ ceramics etched with barrier discharge plasma (DBD).

**Figure 8:**
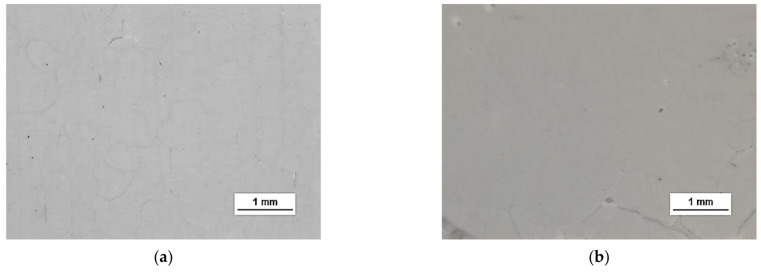
Microscope images of the surface of graphene: (**a**) HSMG^®^ and (**b**) CVD, transferred to Al_2_O_3_ ceramics etched with O_2_ plasma using RF PACVD method.

**Figure 9:**
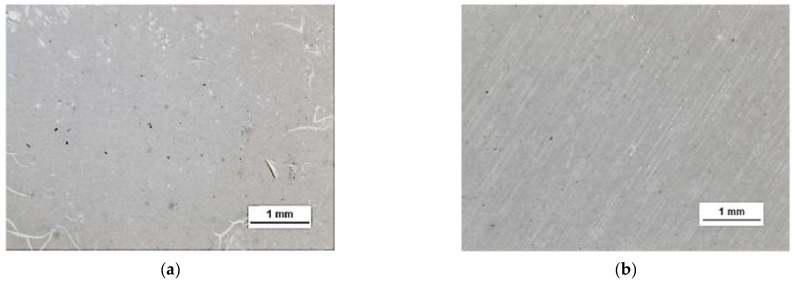
Microscope images of the surface of graphene (**a**) HSMG^®^ and (**b**) CVD, transferred to Al_2_O_3_ ceramics etched with 9.5% HF acid.

HSMG^®^ and CVD graphene sheets were transferred onto surface-modified (O_2_ plasma, DBD, and 5% HF etching) ceramic substrates using the wet transfer method supported by a PDMS/PMMA layer. Then, a qualitative assessment of graphene on an aluminium oxide substrate was carried out. Images obtained with a stereomicroscope, at 40× magnification, showed the surface of both types of graphene after etching the ceramics with DBD barrier discharge plasma (Figure 7), RF PACVD oxygen plasma (Figure 8), and 5% hydrofluoric acid (Figure 9).

Observation of the surface of the graphene transferred onto the modified ceramics on a macro scale showed that the plasma modification of the ceramic surface improved the quality of the graphene after the transfer to a greater extent than chemical modification. Transferred sheets of HSMG^®^ and CVD graphene onto the plasma-modified surface of Al_2_O_3_ ceramics showed good continuity and uniformity of coverage. However, graphene after transfer to the chemically etched surface showed numerous macroscopic damages, but the nature of these damages depended on the method of graphene production. HSMG^®^ graphene had single-point discontinuities and short cracks. In contrast, CVD graphene transferred to the same substrate had uniform, parallel linear discontinuities. There are no such damages on the surface of HSMG^®^ and CVD graphene after transfer to ceramics modified with the oxygen and barrier plasmas.

**Figure 10:**
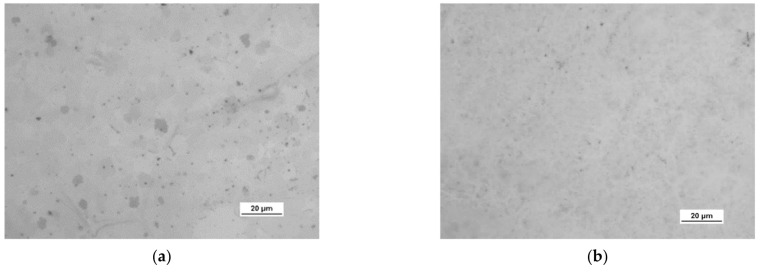
Optical images of the surface microstructure of: (**a**) HSMG^®^ and (**b**) CVD graphene transferred to O_2_ RF PACVD plasma-etched Al_2_O_3_ ceramics.

**Figure 11:**
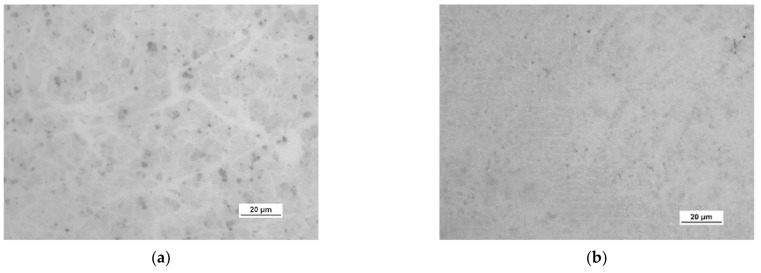
Optical images of the surface microstructure of (**a**) HSMG^®^ and (**b**) CVD graphene transferred to barrier discharge plasma (DBD)-etched Al_2_O_3_ ceramics.

**Figure 12:**
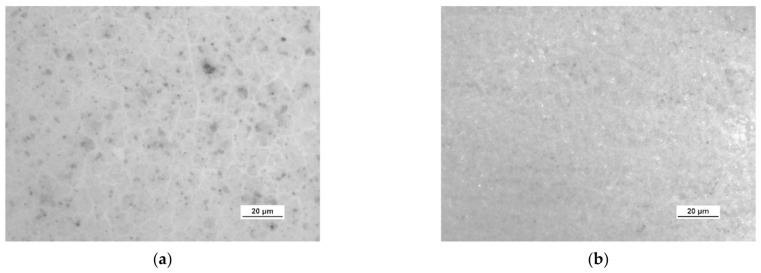
Optical images of the surface microstructure of: (**a**) HSMG^®^ and (**b**) CVD graphene transferred to 5% HF acid-etched Al_2_O_3_ ceramics.

A more detailed assessment of the degree of microstructure defects of the transferred HSMG^®^ and CVD graphene layers after plasma and chemical modification of alumina ceramics (Al_2_O_3_) was carried out under a light microscope. The analysis of the images showed that cleaning the ceramic substrate with oxygen plasma (Figure 10) allowed the transfer of a much less defective HSMG^®^ graphene layer than after etching the ceramic with barrier discharge plasma (Figure 11) and HF acid (Figure 12). In the case of surface modification with DBD plasma and chemical etching, the transferred HSMG^®^ graphene was characterized by a large number of cracks of different sizes. In the case of CVD graphene, no such defects were found, although after transferring this graphene to the chemically modified surface, numerous point defects were observed. The HSMG^®^ graphene was also slightly more contaminated than CVD graphene. This was most obviously related to the degree of purity of the fabrication process. 

Table 3 presents the statistical data on the assessment of the degree of defects in graphene after transfer to ceramics. Using the image analysis program, the total area of discontinuities (defects) per 1 cm^2^ of graphene surface was estimated. For HSMG^®^ graphene, the most defects were visible after HF etching, and the best result was obtained for O_2_ plasma treatment. CVD graphene, on the other hand, had the best quality after applying DBD, but the result after surface modification with oxygen plasma was similar.

### 3.4. Quality Assessment of HSMG^®^ and CVD Graphene after Transfer to Surface-Modified Ceramics Using Raman Spectroscopy

**Figure 13:**
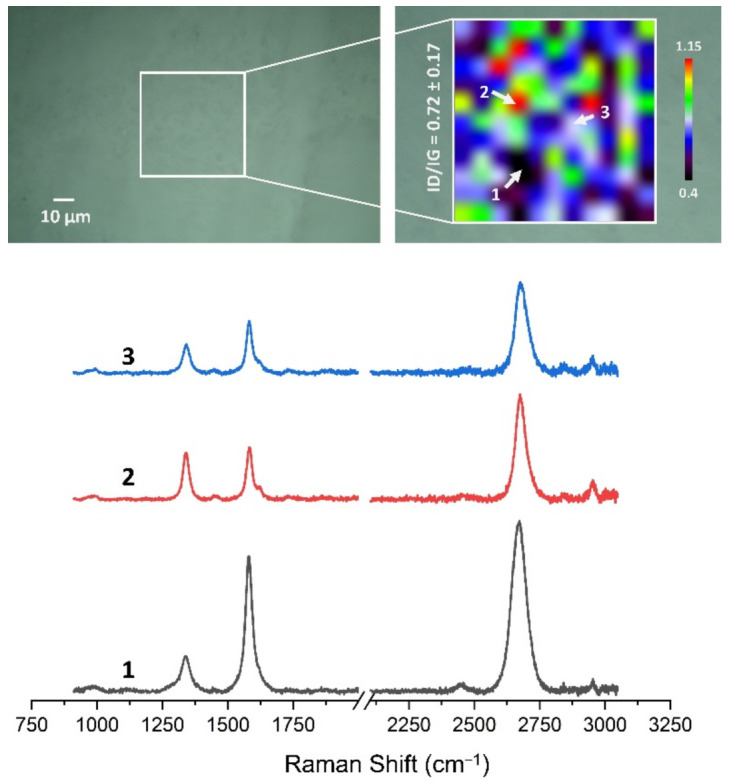
The Raman map and spectra of HSMG^®^ graphene transferred onto Al_2_O_3_ ceramic substrates modified by DBD plasma.

**Figure 14:**
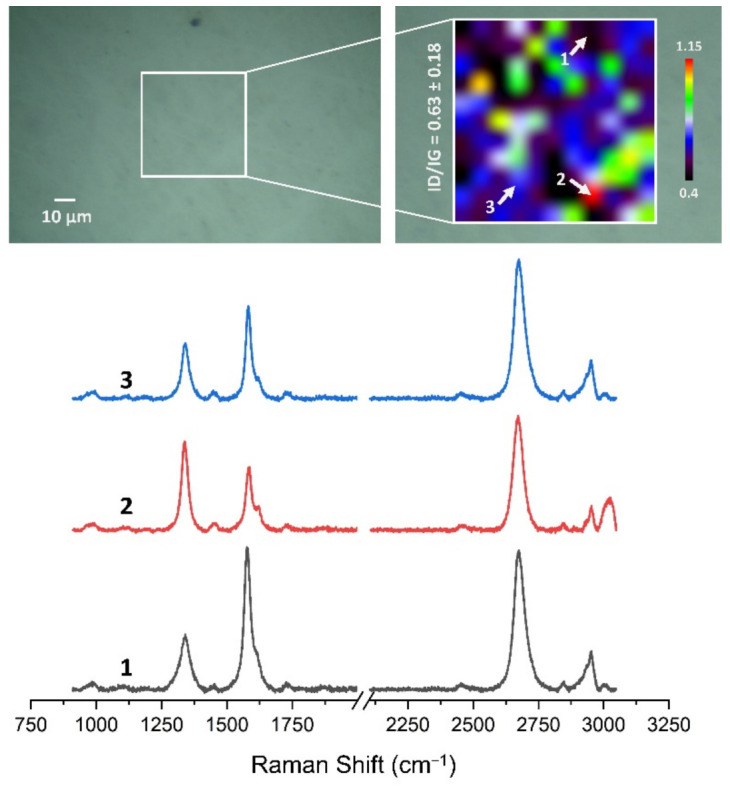
The Raman map and spectra of HSMG^®^ graphene transferred onto Al_2_O_3_ ceramic substrates modified by RF PACVD oxygen plasma.

**Figure 15:**
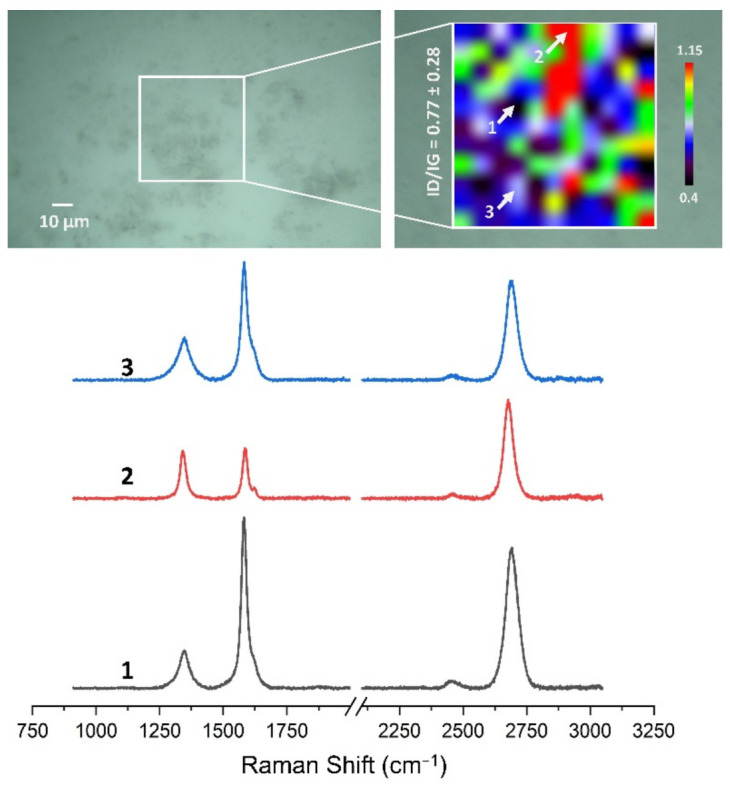
The Raman map and spectra of HSMG^®^ graphene transferred onto Al_2_O_3_ ceramic substrates etched with 5% HF acid.

**Figure 16:**
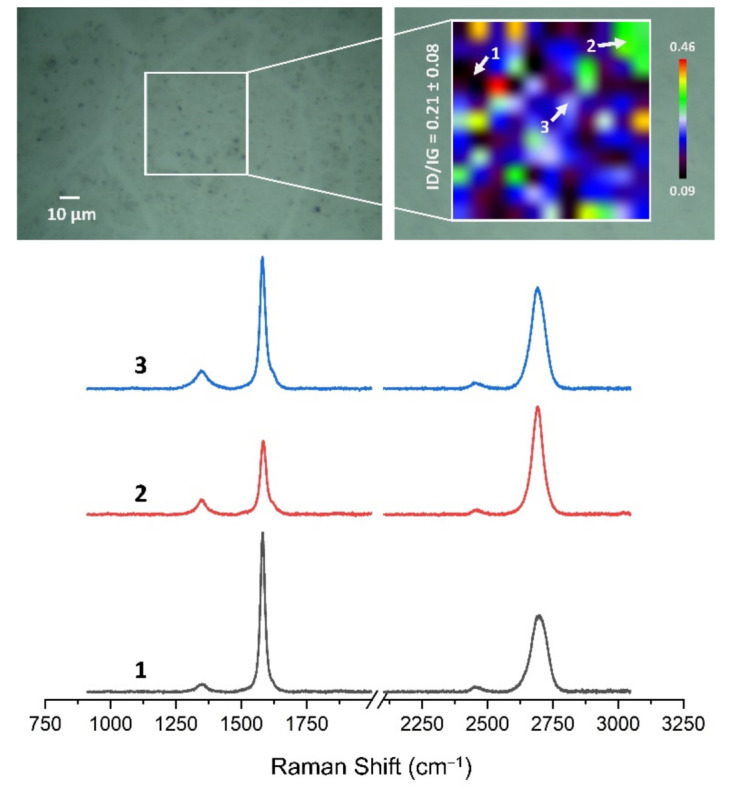
The Raman map and spectra of CVD graphene transferred onto Al_2_O_3_ ceramic substrates modified by DBD plasma.

**Figure 17:**
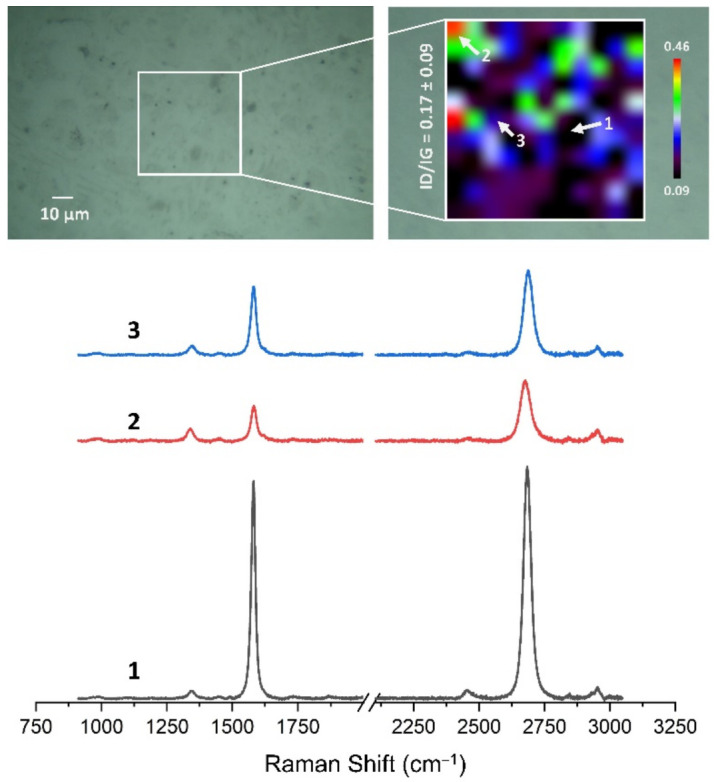
The Raman map and spectra of CVD graphene transferred onto Al_2_O_3_ ceramic substrates modified by RF PACVD oxygen plasma.

**Figure 18:**
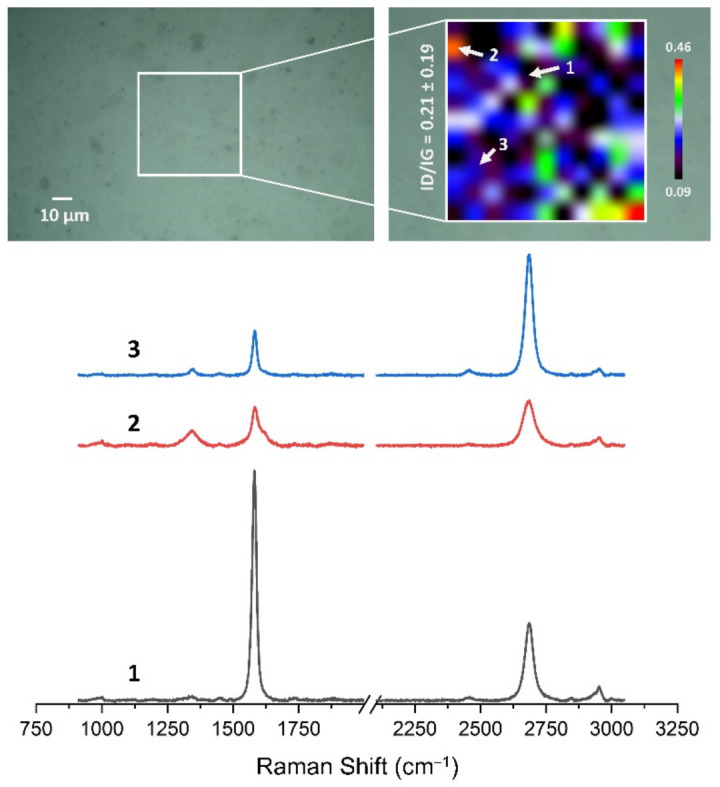
The Raman map and spectra of CVD graphene transferred onto Al_2_O_3_ ceramic substrates etched with 5% HF acid.

Figure 13, Figure 14, Figure 15, Figure 16, Figure 17 and Figure 18 show Raman maps of HSMG^®^ and CVD graphene transferred onto Al_2_O_3_ ceramic substrates modified by the three different methods. The spectra presented are typical of the graphene [7,11,13,31,33,48,49] with prominent D (at about 1340 cm^−1^), G (at about 1580 cm^−1^), and 2D (about 2690 cm^−1^) peaks. The presented maps show the change in the ratio of D to G peaks on the studied surfaces, which, according to the literature [11,49,50,51], are indicative of changes in the degree of graphene defects after transfer to surface-modified Al_2_O_3_ ceramics. On the studied surfaces, the lowest, highest, and intermediate degree of defect sites with the lowest (marked “1”), highest (marked “2”), and intermediate (marked “3”) I_D_/I_G_ ratio values are indicated. The least damaged layer of HSMG^®^ graphene was obtained after transfer to ceramics modified by oxygen plasma (RF PACVD). The most defective HSMG^®^ graphene was on ceramics etched with 5% HF. The same was true for CVD graphene. The least damaged graphene was found on ceramics modified with oxygen plasma and a similar result was obtained for the barrier plasma. The degree of damage to the graphene layer on the substrate etched with 5% HF was the highest.

On the basis of the analysis carried out for each sample, the average value of the I_D_/I_G_ ratio was also determined for all points from the tested surfaces. When studying the transferred HSMG^®^ graphene, the average I_D_/I_G_ ratio was shown to be the lowest for the initial modification of substrates with RF PACVD oxygen plasma (0.63 ± 0.18). When DBD plasma was used, it increased to a value of 0.72 ± 0.17, and in the case of ceramic surface treatment with acids, it was already 0.77 ± 0.28. On the other hand, in the study of CVD graphene, the lowest I_D_/I_G_ values were obtained for modification with DBD plasma (0.17 ± 0.09), while for samples treated with RF PACVD oxygen plasma or treated with acids, the values were higher and were 0.21 ± 0.08 and 0.21 ± 0.19, respectively.

## 4. Conclusions

The presented work proves that by modifying the surface properties of Al_2_O_3_ ceramics, it is possible to transfer monolayer HSMG^®^ and CVD graphene to this substrate. The surface modification strongly affects the change of the free energy of the ceramic surface. This surface, especially after plasma modification, became strongly hydrophilic. This had a direct impact on the possibility of transferring graphene sheets. The quality of the transferred graphene, in turn, is affected by the modification method used. The conducted tests showed that the best quality HSMG^®^ graphene layers were the ones transferred to a substrate modified with low-temperature oxygen plasma (RF PACVD). Additionally, for CVD graphene, the best option to modify the surface of ceramics was barrier plasma (DBD). As indicated by the FTIR research, this results from the chemical cleaning of the surface, consisting of the removal of organic impurities, and most of the hydroxyl groups are related to the chemical activation of this surface. FTIR studies of ceramics modified with barrier plasma did not confirm such surface changes as was the case with oxygen plasma. This may be related to the shorter duration of the modification effect in the case of DBD, which did not allow the study of this effect on FTIR. Qualitatively, both HSMG^®^ and CVD graphene were characterized by a low degree of defects after modification with oxygen or barrier plasma. Large damage to the graphene layers, both on a macroscopic and microscopic scale, was observed for samples subjected to chemical modification. This is probably due to the different nature of these changes, which in this case are related to the development of the ceramic surface. A slight effect of the graphene production method on its quality after transfer to ceramics was also found. Research indicates that graphene produced by CVD in this case was characterized by a slightly lower defect degree after transfer to Al_2_O_3_. It should be emphasised that the degree of defects in graphene layers is not only related to transfer. Damage to the graphene layers arises first during its synthesis, and again during the transfer. Based on both microscopic and Raman spectroscopy studies (the probed D band), the source of origin cannot be unequivocally indicated.

In conclusion, the obtained research results allow for obtaining composite membranes based on graphene and Al_2_O_3_ ceramics. The preparation of such novel composites could find practical applications as stable membrane materials for the filtration of liquids and gases at extreme temperatures and pH or under high pressure in many industries, such as the biotechnology, pharmaceutical, food, chemical, and petrochemical industries. However, for their practical use in filtration, further research is needed to assess their effectiveness. This work focused primarily on the development of an effective method of transferring large-area graphene to a porous ceramic substrate with Al_2_O_3_ and assessing its quality after transfer on a macro- and microscopic scale. Further research should include the assessment of graphene defects on the ceramic substrate at the nanoscale and the determination of the filtration characteristics of these membranes.

## Figures and Tables

**Table 1:** Parameters of the process of etching the surface of ceramics with oxygen plasma by the RF PACVD method.

Pressure [Pa]	Bias Voltage [V]	Time [Min]	Flow O_2_ [sccm]
22	−100	5	20

**Table 2:** Results of wetting angle measurements for distilled water on modified Al_2_O_3_ surfaces.

Modification Type		$\mathrm{Average}\;\mathrm{Contact}\;\mathrm{Angle},\;\bar{\theta }\;[\text{⸰}]$
Reference sample		51.2 ± 0.65
DBD		outside measuring range (superhydrophilic surface)
Plasma O_2_		
HF acid etching	5%	<5
	9.5%	<5

**Table 3:** Graphene defects analysis based on optical microscope images.

	Graphene Defect Surface Area after Transfer to Al_2_O_3_ [mm^2^/cm^2^]		
	DBD	O_2_ plasma	HF etching
HSMG^®^	1.146	0.251	2.955
CVD	0.083	0.094	0.146

## Data Availability

Not applicable.
